# Supplementary material for: Splice-Junction-Based Mapping of Alternative Isoforms in the Human Proteome
Source: Cell Rep. Author manuscript; Available in PMC 2020 Jan 15. (PMC6961840; doi:10.1016/j.celrep.2019.11.026)
Supplement: 3 [file NIHMS1546469-supplement-3.zip › DF2/PXD006675/AtrialSeptum-151-Q13332-SGALQIESSEETDQGK.pdf]

A

Predicted sequence disorder and sequence features of Q13332

Peptide: SGALQIESSEETDQGK Junction: sp|Q13332|PTPRS_HUMAN|ENSG00000105426|SE2|1805|chr19|5258127|5262972|−2|r45|T1 TrNovel: FALSE

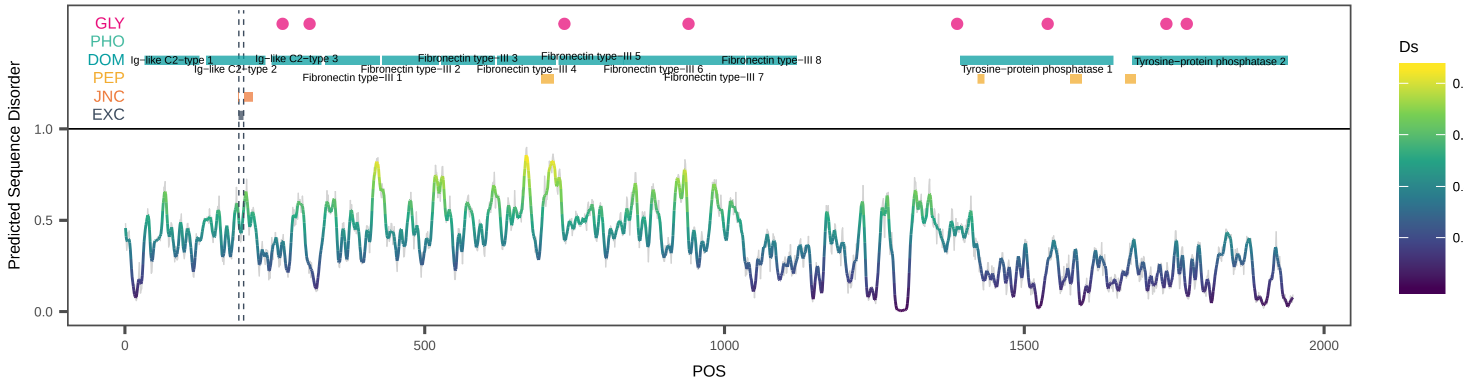

B

Distribution of sequence disorder in excised vs. mapped and non−excised regions of protein

M−W P−value vs. mapped: 0.0302 vs. non−excised: 0.0235

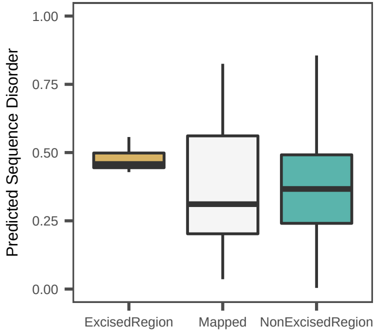

C
